# DNAJB9 in Fibrillary Glomerulonephritis: Diagnostic Biomarker, Putative Autoantigen, or Disease-Associated Scaffold? A Case-Based Narrative Review

**DOI:** 10.3390/life16071186

**Published:** 2026-07-17

**Authors:** Larrisa Lebedev, Mahmud Mansur, Mustafa Seh, Elena Rotshild, Ornit Itzhaki, Alexander Wechsler, Anna Tobar, Nomy Levin Iaina

**Affiliations:** 1 Department of Nephrology and Hypertension, Barzilai University Medical Center, Ashkelon 7830604, Israel; larisal@bmc.gov.il (L.L.); mahmoodm@bmc.gov.il (M.M.); mustafas@bmc.gov.il (M.S.); elenar@bmc.gov.il (E.R.); oraniti@bmc.gov.il (O.I.); alexanderw@bmc.gov.il (A.W.); 2 School of Medicine, Faculty of Health Sciences, Ben Gurion University of the Negev, Beer Sheva 8410501, Israel; 3 Department of Pathology, Rabin Medical Center, Petah Tikva 4941492, Israel; anato@clalit.org.il

**Keywords:** fibrillary glomerulonephritis, DNAJB9, autoantigen, fibril formation, unfolded protein response, crescentic glomerulonephritis, proteinuria, kidney biopsy

## Abstract

Fibrillary glomerulonephritis (FGN) is an uncommon glomerular deposition disease characterized by randomly oriented, nonbranching fibrils that are usually Congo-red-negative and larger than amyloid fibrils on electron microscopy. The discovery of DNAJ homolog subfamily B member 9 (DNAJB9) has transformed FGN from a primarily ultrastructural diagnosis into a molecularly recognizable disease. However, the pathogenic significance of DNAJB9 remains unresolved: it may represent a highly specific biomarker, a putative autoantigen, a chaperone-associated scaffold, or a marker of disturbed protein quality control. We report two patients with positive glomerular DNAJB9 staining and markedly divergent clinical phenotypes. The first patient, a 58-year-old man, presented with severe acute kidney injury, nephritic urinary sediment, severe hypertension, crescentic immune-complex glomerulonephritis, and dialysis-requiring kidney failure. Electron microscopy and IgG subclass staining were unavailable; therefore, the findings were interpreted as probable rather than definitive DNAJB9-positive FGN. Diagnostic and causal interpretation was further complicated by concurrent *Klebsiella pneumoniae* urinary tract infection, mild bilateral hydronephrosis, acute tubulointerstitial injury, and severe hypertension. Kidney function did not recover despite glucocorticoids and cyclophosphamide, but the adverse outcome and treatment response cannot be attributed to FGN alone. The second patient, a 66-year-old woman, presented with chronic proteinuria, preserved kidney function, inactive urinary sediment, and lupus-like serologic findings. Electron microscopy demonstrated randomly arranged, nonbranching 13–19 nm fibrils, and DNAJB9 staining confirmed FGN. She was managed with angiotensin receptor blockade and dapagliflozin, with reduction of proteinuria to below 1 g/day. Together with previously published cohorts, these cases illustrate the diagnostic value of DNAJB9 staining and the potential clinicopathologic heterogeneity of FGN. However, Case 1 should be considered a clinically confounded, hypothesis-generating example and not definitive evidence that FGN alone caused the crescentic presentation, dialysis dependence, or lack of response to immunosuppression.

## 1. Introduction

Fibrillary glomerulonephritis (FGN) is a rare glomerular disease reported in approximately 0.4–1.5% of native kidney biopsies. It is characterized by glomerular deposition of randomly oriented, nonbranching fibrils that are usually Congo-red-negative and commonly measure approximately 12–30 nm, with many series reporting a mean diameter near 20 nm. By routine immunofluorescence, deposits often contain IgG, C3, and both kappa and lambda light chains, supporting an immune-complex component and creating diagnostic overlap with membranoproliferative, membranous-like, lupus-like, infection-related, C3-dominant, and monoclonal immunoglobulin-associated glomerulonephritides [1,2,3,4].

The identification of DNAJ homolog subfamily B member 9 (DNAJB9) as an abundant and highly specific protein within FGN deposits was a major conceptual advance. Laser microdissection with mass spectrometry identified DNAJB9 as a dominant component of FGN glomerular deposits, and subsequent DNAJB9 immunohistochemistry showed very high sensitivity and specificity for FGN [5,6,7,8]. DNAJB9 staining can confirm the diagnosis in limited samples, atypical histologic patterns, or settings where electron microscopy is unavailable, although optimal diagnosis still integrates light microscopy, immunofluorescence, Congo red staining, DNAJB9 staining, and ultrastructural findings.

Yet DNAJB9 is more than a diagnostic stain. DNAJB9, also known as ERdj4, is an endoplasmic reticulum-associated Hsp40/DNAJ co-chaperone involved in protein folding, ER stress responses, and ER-associated degradation [7,9,10]. Its selective accumulation within glomerular fibrils raises a central unresolved question: is DNAJB9 the initiating antigen, a structural scaffold, a secondary binding partner of abnormal immunoglobulin, or a downstream marker of a broader protein misfolding process? This question is not only mechanistic. It may also help explain why patients with the same DNAJB9-positive diagnosis can present with dramatically different clinical and histologic patterns.

Here, we present one electron microscopy-confirmed case of DNAJB9-positive FGN with chronic, proteinuric and preserved kidney function and one DNAJB9-positive crescentic immune-complex glomerulonephritis interpreted as probable FGN in the absence of ultrastructural confirmation. We frame the cases around the pathogenesis of DNAJB9-associated fibril formation and discuss how a shared molecular signature may be associated with clinically divergent disease.

## 2. Case Presentations

### 2.1. Case 1: DNAJB9-Positive Crescentic Immune-Complex Glomerulonephritis, Interpreted as Probable FGN, Presenting as Dialysis-Requiring Kidney Failure

A 58-year-old man presented in November 2025 with one week of nausea, vomiting, generalized weakness, edema, and severe hypertension. Two months earlier, he had received amoxicillin/clavulanate for a dental problem. He had no known systemic autoimmune disease or family history of kidney disease. Four months before presentation, serum creatinine was 1.39 mg/dL, corresponding to an estimated glomerular filtration rate (eGFR) of 59 mL/min/1.73 m^2^; earlier creatinine values were reportedly normal. A urinalysis in 2022 had shown microscopic hematuria and low-grade proteinuria without further evaluation.

On admission, blood pressure was 200/125 mmHg. Laboratory testing showed severe kidney dysfunction and metabolic acidosis, with serum creatinine 6.56 mg/dL, urea 137 mg/dL, potassium 5.39 mmol/L, hemoglobin 10.8 g/dL, and bicarbonate 18 mmol/L. Urinalysis showed proteinuria, hematuria, and leukocyturia. Quantitative urine testing demonstrated an albumin-to-creatinine ratio of 2058 mg/g, protein-to-creatinine ratio of 3141 mg/g, and 24-h protein excretion of 2481 mg/day. Urine microscopy showed marked leukocyturia, erythrocytes including some dysmorphic red cells, granular casts, and one leukocyte cast. Urine culture grew *Klebsiella pneumoniae*, and non-contrast computed tomography demonstrated swollen kidneys with mild bilateral hydronephrosis.

Serologic evaluation showed positive ANA with a speckled pattern at 2+, whereas anti-dsDNA, anti-GBM, anti-PLA2R, C-ANCA, P-ANCA, antiphospholipid antibodies, HIV, and hepatitis B testing were negative. Complement levels were normal, and serum protein electrophoresis and immunofixation were reported as normal. The differential diagnosis included immune-complex rapidly progressive glomerulonephritis, lupus-like nephritis, infection-associated GN, pauci-immune or anti-GBM crescentic GN, malignant hypertension-associated kidney injury, obstruction-related injury, and possible drug-associated interstitial nephritis.

Despite blood pressure control, treatment of urinary tract infection, and supportive care, kidney function deteriorated, with serum creatinine rising to 9.47 mg/dL and urea to 282 mg/dL. Hemodialysis was initiated via a tunneled dialysis catheter.

**Figure 1:**
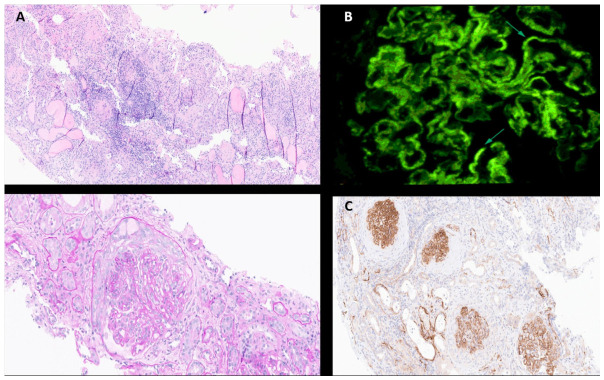
Renal biopsy findings in Case 1. (**A**) Light microscopy shows renal cortical tissue with crescentic glomerular injury, tubular damage, and a lymphoplasmacytic-predominant interstitial inflammatory infiltrate. Upper left panel: hematoxylin and eosin stain, original magnification ×40. Lower left panel: periodic acid–Schiff stain, original magnification ×200, demonstrating a glomerulus with crescent formation and architectural distortion. (**B**) Immunofluorescence microscopy for IgG shows staining in mesangial and capillary wall distributions. (**C**) DNAJB9 immunohistochemistry demonstrates positive glomerular staining, supporting a probable diagnosis of DNAJB9-positive fibrillary glomerulonephritis.

A kidney biopsy was performed in November 2025 (Figure 1). Light microscopy showed renal cortical tissue containing 20 glomeruli. Four glomeruli demonstrated segmental or global endocapillary/extracapillary proliferation, and 11 glomeruli showed cellular and fibrocellular crescents; karyorrhexis was present in some glomeruli. Seven glomeruli showed ischemic changes or global sclerosis. There was distortion of the glomerular architecture, tubular damage with dilatation, and a lymphoplasmacytic-predominant interstitial inflammatory infiltrate (Figure 1A). Congo red staining was negative. Immunofluorescence showed immune-complex staining for IgG and C3 in mesangial and capillary wall distributions, C1q staining, and both kappa and lambda light chain staining (Figure 1B). IgG subclass staining was not performed. DNAJB9 immunostaining was positive in glomerular deposits (Figure 1C).

Electron microscopy could not be performed because insufficient biopsy material remained for ultrastructural evaluation, and IgG subclass staining was not available. Positive glomerular DNAJB9 staining, Congo red negativity, and polytypic IgG, C3, C1q, kappa, and lambda staining strongly favored FGN; however, in the absence of direct demonstration of randomly arranged nonbranching fibrils, we regard this as a probable or working diagnosis of DNAJB9-positive FGN rather than a definitive diagnosis.

Lupus nephritis and lupus-like immune-complex GN were considered because of the positive ANA and C1q staining; however, anti-dsDNA was negative, complement levels were normal, and there were no documented systemic clinical features of lupus. Pauci-immune crescentic GN and anti-GBM disease were considered less likely because ANCA and anti-GBM antibodies were negative and immune-complex staining was present. Infection-associated GN, malignant hypertension-associated injury, obstruction-related injury, and possible drug-associated interstitial nephritis were also considered as potential contributors to the acute kidney injury, particularly given the Klebsiella urinary tract infection, severe hypertension, mild bilateral hydronephrosis, recent antibiotic exposure, and acute tubulointerstitial injury.

However, these factors did not fully explain the DNAJB9-positive crescentic immune-complex glomerular pattern. Thus, the renal presentation in Case 1 was interpreted as multifactorial. The crescentic DNAJB9-positive immune-complex glomerular lesion provided evidence of an active glomerular disease process, whereas *Klebsiella pneumoniae* urinary tract infection, severe hypertension, mild bilateral hydronephrosis, recent antibiotic exposure, acute tubulointerstitial injury, and severe metabolic derangements likely contributed to the severity of AKI and to the need for dialysis. Because these processes occurred simultaneously, their relative contributions to the final dialysis-dependent outcome cannot be quantified from this single case.

Given the rapidly progressive course and the crescentic immune-complex pattern, the patient received pulse intravenous methylprednisolone 1 g daily for three consecutive days, followed by oral prednisone 80 mg/day with a gradual taper. In parallel, he received three doses of intravenous cyclophosphamide, 900 mg per dose. Despite aggressive immunosuppressive therapy, kidney function did not recover, and the patient remained dialysis-dependent on maintenance hemodialysis. However, the absence of renal recovery cannot be attributed solely to the severity of FGN or to lack of response to immunosuppression, because the clinical course was also complicated by severe hypertension, *Klebsiella pneumoniae* urinary tract infection, acute tubulointerstitial injury, and mild bilateral hydronephrosis. These factors may have contributed to the dialysis-requiring kidney failure and limited the ability to determine the relative contribution of crescentic DNAJB9-positive FGN to the final renal outcome.

### 2.2. Case 2: DNAJB9-Positive FGN Presenting as Chronic Proteinuric Disease with Preserved Kidney Function

A 66-year-old woman with hypothyroidism and hyperlipidemia was followed by a nephrologist for persistent proteinuria. In October 2020, the albumin-to-creatinine ratio was 2508 mg/g, and valsartan 80 mg daily was initiated. Albuminuria subsequently improved to 812 mg/g in February 2021. In July 2021, the albumin-to-creatinine ratio was 611 mg/g, the protein-to-creatinine ratio was 2000 mg/g, serum creatinine was 0.47 mg/dL, and eGFR was 105 mL/min/1.73 m^2^. In 2023, microscopic urinalysis was negative for leukocytes and erythrocytes. In February 2024, proteinuria worsened, and dapagliflozin was added to ongoing angiotensin receptor blocker (ARB) therapy. Under supportive nephroprotective treatment with an ARB and dapagliflozin, proteinuria decreased to below 1 g/day, kidney function remained preserved, and no immunosuppressive therapy was administered. Complement levels were normal. The immunologic workup showed positive anti-dsDNA and borderline ANA antibodies; thyroglobulin antibody was elevated. Testing for hepatitis B, hepatitis C, anti-PLA2R antibodies, serum protein electrophoresis, immunofixation, and serum free light chains were negative or normal.

**Figure 2:**
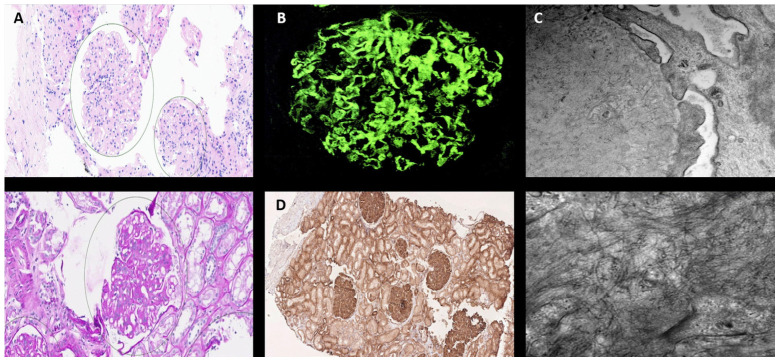
Renal biopsy findings in Case 2. (**A**) Light microscopy shows glomerular basement membrane thickening, mesangial matrix expansion, and mild mesangial hypercellularity, without endocapillary hypercellularity, necrosis, or crescent formation. Upper left panel: hematoxylin and eosin stain, original magnification ×100. Lower left panel: periodic acid–Schiff stain, original magnification ×200, highlighting mesangial expansion and capillary wall thickening. (**B**) Immunofluorescence microscopy for IgG shows mesangial and capillary wall staining. (**C**) Electron microscopy demonstrates randomly arranged, nonbranching fibrillary deposits measuring approximately 13–19 nm, consistent with fibrillary glomerulonephritis. Upper right panel: low-power electron microscopy; lower right panel: high-power electron microscopy showing fibrillary deposits. (**D**) DNAJB9 immunohistochemistry demonstrates diffuse positive glomerular staining, establishing the diagnosis of DNAJB9-positive fibrillary glomerulonephritis.

The clinical differential diagnosis included lupus nephritis, amyloidosis, primary or secondary focal segmental glomerulosclerosis, membranous nephropathy, and other rare glomerular diseases. A kidney biopsy was performed in January 2025 (Figure 2). Light microscopy identified 16 glomeruli with glomerular basement membrane thickening, mesangial expansion predominantly due to increased mesangial matrix, and only mild segmental mesangial hypercellularity. No endocapillary hypercellularity, necrosis, or crescents were identified. The interstitium, tubules, and blood vessels were reported as unremarkable (Figure 2A). Congo red staining was negative. Immunofluorescence showed mesangial and capillary wall immune-complex staining with IgG and C3, C1q staining, and both kappa and lambda light chain staining (Figure 2B). IgG subclass staining was not performed. Electron microscopy demonstrated randomly arranged, nonbranching 13–19 nm fibrillary deposits (Figure 2C). DNAJB9 staining was positive (Figure 2D), establishing the diagnosis of FGN.

The presentation was therefore consistent with DNAJB9-positive FGN manifesting as chronic proteinuric disease with preserved kidney function and lupus-like serologic features, but without clinical or biopsy features of classic active lupus nephritis. Lupus nephritis was considered because of the positive anti-dsDNA, borderline ANA, and C1q staining.

However, complement levels were normal, urinary sediment was inactive, and the biopsy did not show endocapillary hypercellularity, necrosis, crescents, or other features of active proliferative lupus nephritis. In addition, electron microscopy demonstrated randomly arranged, nonbranching fibrillary deposits measuring 13–19 nm, and DNAJB9 immunostaining was positive.

These findings favored DNAJB9-positive FGN rather than classic lupus nephritis or nonspecific lupus-like immune-complex GN. When compared with lupus nephritis patterns, the biopsy did not show features supportive of active proliferative lupus nephritis, such as segmental or global endocapillary hypercellularity, necrotizing lesions, cellular crescents, or prominent subendothelial immune-complex deposits. The findings were also not typical of membranous lupus nephritis as the dominant process, because the key ultrastructural abnormality was randomly arranged nonbranching fibrillary deposits rather than a membranous pattern of immune-complex deposition.

Thus, although the positive anti-dsDNA, borderline ANA, and C1q staining created a lupus-like diagnostic context, the combination of inactive urinary sediment, normal complement levels, absence of active lupus nephritis lesions on light microscopy, fibrillary deposits on electron microscopy, and positive DNAJB9 staining favored DNAJB9-positive FGN over lupus nephritis with fibrillary deposits or nonspecific lupus-like immune-complex GN.

The reduction in proteinuria during treatment with angiotensin receptor blockade and dapagliflozin was interpreted as an encouraging individual response to general nephroprotective therapy. However, this single case does not establish a disease-specific therapeutic effect of SGLT2 inhibition in FGN, nor does it support a general treatment recommendation for DNAJB9-positive FGN with a proteinuric phenotype. In addition, the follow-up duration was limited, and no repeat biopsy was performed; therefore, the reduction in proteinuria cannot be interpreted as evidence of regression or modification of DNAJB9-positive fibrillary deposits.

The comparative clinical, serologic, pathologic, therapeutic, and follow-up features of the two cases are summarized in Table 1.

## 3. Discussion

### 3.1. From Morphologic Disease to Molecular Disease

Before DNAJB9, FGN was defined by morphology: randomly arranged fibrils within the mesangium and/or glomerular basement membranes, larger than amyloid and typically Congo-red-negative. This definition explained the biopsy appearance but did not identify the molecular driver. Proteomic studies changed this paradigm by showing that DNAJB9 is enriched in FGN deposits and is not similarly enriched in amyloidosis, immunotactoid glomerulopathy, or other organized deposit diseases. DNAJB9 immunostaining subsequently became a practical diagnostic tool with reported sensitivity around 98% and specificity around 99% in major validation studies [6,7,8,11].

### 3.2. Differential Diagnosis with Lupus Nephritis and Other Immune-Complex Glomerulonephritides

A central diagnostic challenge in both cases was the overlap between DNAJB9-positive FGN and other immune-complex glomerulonephritides. FGN often shows glomerular IgG and C3 deposition with polytypic light chain staining, and in some cases may show C1q staining or lupus-like features, creating potential overlap with lupus nephritis, membranous nephropathy, membranoproliferative GN, infection-associated GN, and monoclonal immunoglobulin-associated glomerular disease. Therefore, the presence of immune-complex staining or autoimmune serologies alone should not be considered diagnostic of lupus nephritis without supportive clinical, serologic, and pathologic findings.

In Case 1, lupus nephritis was considered because of the positive ANA and C1q staining, but anti-dsDNA was negative, complement levels were normal, and no systemic lupus features were documented. The biopsy showed a crescentic immune-complex pattern with polytypic staining and positive DNAJB9 immunostaining. Although electron microscopy was not available, the negative Congo red stain, positive DNAJB9 staining, and exclusion of major alternative causes supported DNAJB9-positive FGN.

Infection-associated GN, malignant hypertension-associated injury, obstruction-related injury, and drug-associated interstitial nephritis were also considered, particularly given the Klebsiella urinary tract infection, severe hypertension, mild bilateral hydronephrosis, recent antibiotic exposure, and acute tubulointerstitial injury. These conditions may have contributed to the severity of AKI, but they did not fully account for the DNAJB9-positive crescentic immune-complex glomerular lesion.

In Case 2, the positive anti-dsDNA, borderline ANA, and C1q staining raised the possibility of lupus nephritis or lupus-like immune-complex GN. However, complement levels were normal, urinary sediment was inactive, and the biopsy lacked active proliferative features such as endocapillary hypercellularity, necrosis, or crescents. The presence of randomly arranged, nonbranching fibrillary deposits on electron microscopy together with positive DNAJB9 staining favored FGN over classic active lupus nephritis. Thus, the case was interpreted as DNAJB9-positive FGN with lupus-like serologic features rather than lupus nephritis as the primary diagnosis.

IgG subclass staining was not available in either case. This is relevant because IgG subclass distribution may provide additional diagnostic context in immune-complex glomerulonephritides, including lupus nephritis, membranous nephropathy, infection-associated GN, monoclonal immunoglobulin-associated disease, and FGN.

Its absence limited further characterization of the immune deposits. Nevertheless, in Case 2, the presence of randomly arranged nonbranching fibrillary deposits on electron microscopy and positive DNAJB9 staining favored FGN despite the lupus-like serologic background. In Case 1, the absence of both electron microscopy and IgG subclass staining reduced diagnostic certainty, but the diagnosis remained supported by positive glomerular DNAJB9 staining, Congo red negativity, polytypic immunofluorescence, and the overall clinicopathologic context.

The discovery of DNAJB9 also raised an important, but still unresolved, pathogenic question: why does an ER-associated co-chaperone involved in protein homeostasis accumulate within extracellular glomerular fibrils? Current evidence supports DNAJB9 as a highly specific disease-associated protein in FGN, but whether it is an initiating antigen, a structural component of deposits, a secondary binding partner, or a marker of disturbed protein handling remains uncertain. The present cases cannot establish causality; rather, they provide a clinical framework for discussing how DNAJB9-positive FGN may present across a broad clinicopathologic spectrum.

**Figure 3:**
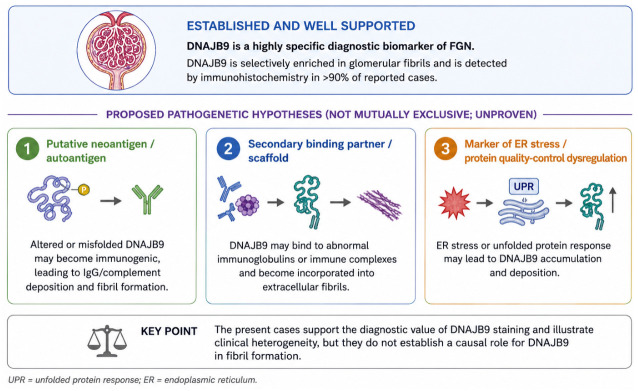
Established diagnostic role and proposed pathogenetic hypotheses for DNAJB9 in FGN. DNAJB9 is well established as a highly specific diagnostic marker of FGN and is selectively enriched within FGN glomerular deposits. The schematic summarizes three non-mutually exclusive pathogenetic hypotheses proposed in the literature, which remain unproven and should not be interpreted as equally established mechanisms. First, DNAJB9 may act as a putative neoantigen or autoantigen, potentially participating in immune recognition, IgG/complement deposition, and fibril formation. Second, DNAJB9 may function as a secondary binding partner or scaffold for abnormal immunoglobulins or immune complexes, becoming incorporated into extracellular fibrillary deposits. Third, DNAJB9 accumulation may reflect dysregulated ER stress, unfolded protein response, or protein quality-control pathways. The present cases support the diagnostic value of DNAJB9 staining and illustrate clinical heterogeneity, but they do not establish a causal role for DNAJB9 in fibril formation.

Because the strength of evidence differs across these concepts, Figure 3 is intended to distinguish the established diagnostic role of DNAJB9 from proposed pathogenetic hypotheses. The selective enrichment of DNAJB9 in FGN deposits and the diagnostic utility of DNAJB9 immunostaining are well supported. In contrast, the roles of DNAJB9 as a causal autoantigen, secondary binding partner or scaffold, or mediator/marker of ER stress and protein quality-control dysregulation remain unresolved. Three non-mutually exclusive hypotheses have been proposed in the literature and are summarized as a conceptual framework rather than as demonstrated mechanisms.

First, DNAJB9 may act as a putative autoantigen. DNAJB9 is abundant in FGN deposits, co-localizes with IgG, and has been localized to fibrillary structures by immunoelectron microscopy. Andeen and colleagues proposed DNAJB9 as a putative autoantigen and suggested that IgG1 and classical complement pathways may contribute to glomerular injury [7]. In this framework, altered or misfolded DNAJB9 could become immunogenic and participate in immune-complex deposition. However, a disease-defining circulating anti-DNAJB9 autoantibody has not become an established clinical test, and deposit-associated DNAJB9 does not by itself prove that DNAJB9 initiates the disease.

Second, DNAJB9 may function as a secondary binding partner or scaffold for abnormal immunoglobulin-containing deposits. In this model, immune complexes or misfolded immunoglobulins may form first, and DNAJB9 may become incorporated into the deposits through its chaperone-related biology. This possibility is consistent with the immune-complex staining pattern seen in many cases of FGN and with reports of monotypic-appearing, immunoglobulin-negative, and other atypical DNAJB9-positive cases, which suggest that the relationship between DNAJB9 and immunoglobulin deposition is complex [9,12,13,14].

Third, DNAJB9 accumulation may reflect dysregulated protein quality-control pathways. DNAJB9/ERdj4 participates in ER protein folding and ER-associated degradation [10]. Although this biology makes the unfolded protein response an attractive conceptual framework, current evidence argues against a simple model of local glomerular DNAJB9 overproduction. Avasare and colleagues found that glomerular DNAJB9 accumulation was not accompanied by increased glomerular DNAJB9 mRNA expression [15], while serum DNAJB9 levels have been reported to be elevated in patients with FGN [16].

These findings suggest that systemic overexpression, altered processing, extracellular trapping, or impaired clearance may contribute, but the precise mechanism remains unresolved. Thus, the pathogenetic role of DNAJB9 should be interpreted cautiously. In the present report, DNAJB9 serves primarily as a diagnostic and disease-defining marker that unifies two clinically divergent cases. The mechanistic discussion and Figure 3 are hypothesis-generating and are intended to organize current concepts from the literature, not to imply that the three pathways are equally established or directly demonstrated by our cases.

### 3.3. A Three-Axis Interpretive Framework for Clinical Heterogeneity in DNAJB9-Positive FGN

The contrast between the two patients can be interpreted through a three-axis framework rather than a validated predictive model. This framework is derived primarily from published FGN cohorts and is not inferred from the present two cases alone. Case 2 provides a confirmed clinicopathologic example, whereas Case 1 is used only as a cautious illustration because its diagnosis was not ultra structurally confirmed and its clinical outcome was affected by several competing kidney insults.

The first axis is deposit distribution and histologic pattern. FGN deposits may involve the mesangium and/or glomerular capillary walls and may be associated with mesangial, membranous-like, membranoproliferative, diffuse proliferative, crescentic, or sclerosing patterns [4,12,17,18]. Published cohorts have suggested that glomerular pattern, including proliferative or crescentic lesions, may be associated with more aggressive clinical presentation and poorer outcomes [17,18]. In our report, Case 1 showed a crescentic immune-complex GN pattern with 11 cellular/fibrocellular crescents among 20 glomeruli and dialysis-requiring AKI, whereas Case 2 showed mesangial matrix expansion, mild mesangial hypercellularity, glomerular basement membrane thickening, and preserved kidney function.

The second axis is the intensity of immune activation. FGN commonly shows IgG and C3 deposition with polytypic light chain staining, and DNAJB9-positive deposits may overlap with immune-complex patterns [4,7,12]. The degree of complement activation, C1q/C3 staining, inflammatory cell recruitment, and crescent formation may influence whether the biopsy appears relatively indolent or actively inflammatory. In Case 1, C3 and C1q staining, karyorrhexis, endocapillary/extracapillary proliferation, and extensive crescents supported a high-inflammatory phenotype. In Case 2, immune-complex staining and C1q were present, but complement levels were normal, urinary sediment was inactive, and the biopsy lacked endocapillary hypercellularity, necrosis, or crescents, supporting a lower-inflammatory proteinuric phenotype.

The third axis is chronicity and baseline renal reserve. Prior studies have linked outcomes in FGN to baseline kidney function, proteinuria, glomerulosclerosis, interstitial fibrosis/tubular atrophy, and crescentic or proliferative patterns [4,17,18]. In Case 1, the prior eGFR was already reduced, seven glomeruli showed ischemic change or global sclerosis, and the biopsy also showed tubular damage and interstitial inflammation, all in the context of superimposed severe hypertension, infection, and mild obstruction. These features may have contributed to poor renal recovery. In Case 2, kidney function was preserved, the urinary sediment was inactive, and the tubulointerstitium and vessels were reported as unremarkable, consistent with a less destructive clinicopathologic presentation.

Thus, the three axes provide a literature-based framework for considering clinical heterogeneity in FGN. The present cases serve as illustrations of this framework and should not be interpreted as validating it. In particular, Case 1 does not establish that the crescentic presentation or dialysis-dependent outcome was attributable to FGN alone. Rather, the framework helps explain why the same molecular diagnosis may be associated with either dialysis-requiring crescentic/RPGN disease or chronic proteinuric disease with preserved kidney function.

### 3.4. Diagnostic Implications

DNAJB9 staining should be considered when kidney biopsy shows immune-complex GN with atypical or discordant clinical features, organized or suspected fibrillary deposits, membranous-like or MPGN-like patterns, crescentic immune-complex GN without a clear systemic diagnosis, apparent lupus-like features with incomplete clinical correlation, or unexplained proteinuria with mesangial matrix expansion. The diagnosis is strongest when DNAJB9 positivity is integrated with light microscopy, immunofluorescence, Congo red staining, electron microscopy when available, and exclusion of amyloidosis and immunotactoid glomerulopathy.

Importantly, positive DNAJB9 staining may be particularly valuable when electron microscopy is unavailable or limited, as in Case 1, provided that the overall clinicopathologic context is compatible.

### 3.5. Treatment and Prognosis in Light of Pathogenesis

There is no established standard therapy for FGN. Supportive care remains foundational and includes blood pressure control, RAAS blockade, SGLT2 inhibition when otherwise clinically appropriate, treatment of edema and cardiovascular risk, and evaluation for associated autoimmune disease, malignancy, viral hepatitis, and monoclonal gammopathy [19]. The pathogenesis models described above provide a rationale for immunomodulatory strategies in selected patients, but clinical responses remain inconsistent.

Rituximab is the best-studied immunosuppressive strategy. Retrospective data and a small prospective pilot study suggest that rituximab may stabilize kidney function in selected patients, particularly when baseline kidney function is not severely impaired, but effects on proteinuria and long-term outcomes are variable [20,21]. Severe crescentic presentations are often treated empirically with high-dose glucocorticoids, cyclophosphamide, rituximab, or combinations, but outcomes are strongly influenced by baseline eGFR, chronic scarring, and superimposed clinical factors [17,18]. This is consistent with the proposed interpretive framework, while not proving a causal relationship between DNAJB9 biology, immune activation, and clinical outcome. Reducing antibody production may help when ongoing immune activation is dominant, but it may be less effective once irreversible scarring and established fibrillary deposits predominate.

The present cases should therefore be interpreted cautiously. In Case 1, the lack of renal recovery occurred in the setting of crescentic DNAJB9-positive immune-complex GN, but also in the presence of severe hypertension, *Klebsiella pneumoniae* urinary tract infection, acute tubulointerstitial injury, and mild obstruction. Therefore, the final dialysis-dependent outcome cannot be attributed solely to the severity of FGN or to failure of methylprednisolone and cyclophosphamide.

In Case 2, the decrease in proteinuria during ARB and dapagliflozin therapy was encouraging, but it should be viewed as an individual short-term response to general nephroprotective therapy rather than evidence of disease-specific modification of DNAJB9-positive deposits or proof of an FGN-specific benefit of SGLT2 inhibition.

These observations support individualized clinicopathologic assessment and cautious therapeutic interpretation rather than phenotype-specific treatment recommendations based on two cases.

The divergent clinical courses of the present cases illustrate this therapeutic uncertainty. Case 1, which presented with dialysis-requiring DNAJB9-positive crescentic GN, did not recover kidney function despite pulse methylprednisolone, high-dose oral prednisone, and cyclophosphamide, suggesting that severe inflammatory injury, extensive crescent formation, and advanced functional loss may be poorly reversible once established. In contrast, Case 2 had preserved kidney function and proteinuria that decreased to below 1 g/day under ARB and dapagliflozin therapy, supporting a nephroprotective approach in patients with lower inflammatory activity, preserved filtration, and a predominantly proteinuric phenotype.

### 3.6. Limitations

This report has several limitations. Most importantly, electron microscopy could not be performed in Case 1 because insufficient biopsy material remained for ultrastructural evaluation. In addition, IgG subclass staining was not performed in either case. As a result, the diagnosis in Case 1 lacks direct ultrastructural confirmation of randomly arranged nonbranching fibrils, and both cases lack IgG subclass characterization of the immune deposits. The absence of IgG subclass staining limited our ability to further compare the deposits with patterns typically seen in lupus nephritis, membranous nephropathy, infection-associated GN, monoclonal immunoglobulin-associated glomerular disease, and FGN. This limitation is particularly relevant for Case 1 because of its atypical crescentic/RPGN phenotype and lack of EM confirmation. However, the overall clinicopathologic pattern remained supportive of DNAJB9-positive FGN. We therefore interpret Case 1 as probable DNAJB9-positive FGN diagnosed without EM confirmation, while acknowledging that the absence of ultrastructural evaluation and IgG subclass staining limits diagnostic certainty compared with Case 2. The two cases should therefore not be assigned equal diagnostic or evidentiary weight. Case 2 provides a definitive example of DNAJB9-positive FGN, whereas Case 1 remains a probable and clinically confounded example. Case 1 should not be used independently to support conclusions regarding the phenotype, prognosis, pathogenesis, or treatment responsiveness of crescentic FGN.

### 3.7. What These Two Cases Add

The value of this report lies in juxtaposing one ultrastructurally confirmed case of DNAJB9-positive FGN with one clinically severe DNAJB9-positive crescentic immune-complex glomerulonephritis that was interpreted as probable FGN. The latter case does not independently establish a crescentic FGN phenotype. Rather, it highlights the diagnostic challenges that arise when electron microscopy is unavailable and multiple competing causes of acute kidney injury coexist.

The broader clinicopathologic heterogeneity of FGN is supported primarily by published cohorts. The present cases illustrate how DNAJB9 staining may contribute to diagnosis in contrasting clinical settings, while also demonstrating the limitations of relying on DNAJB9 positivity without complete ultrastructural and clinicopathologic correlation. The central message is that DNAJB9 provides substantial diagnostic specificity, while its interpretation must remain dependent on complete clinicopathologic correlation. Case 2 demonstrate concordance between DNAJB9 positivity and ultra structurally confirmed FGN, whereas Case 1 illustrates the potential diagnostic utility and limitations of DNAJB9 staining when electron microscopy is unavailable.

## 4. Conclusions

DNAJB9 is a highly useful disease-associated marker for FGN, but its interpretation should remain integrated with light microscopy, immunofluorescence, Congo red staining, electron microscopy when available, and the overall clinical context. In this report, Case 2 represents ultra structurally confirmed DNAJB9-positive FGN with a chronic proteinuric phenotype and preserved kidney function.

In contrast, Case 1 showed positive glomerular DNAJB9 staining in a crescentic immune-complex glomerulonephritis pattern, but the absence of electron microscopy and IgG subclass staining limits diagnostic certainty. Furthermore, severe hypertension, infection, mild obstruction, and acute tubulointerstitial injury substantially complicated attribution of the dialysis-requiring kidney failure and lack of renal recovery. Case 1 should therefore be regarded as a probable, clinically confounded, and hypothesis-generating presentation rather than definitive evidence that FGN alone caused the crescentic phenotype or adverse outcome.

The clinicopathologic heterogeneity of FGN and the potential pathogenic roles of DNAJB9 are supported primarily by the broader published literature. The present cases illustrate both the diagnostic opportunities and the limitations of DNAJB9 staining in contrasting clinical settings.

## Figures and Tables

**Table 1:** The table compares features of two patients with positive glomerular DNAJB9 staining: a clinically confounded crescentic immune-complex glomerulonephritis interpreted as probable FGN in Case 1, and ultra-structurally confirmed DNAJB9-positive FGN with a chronic proteinuria phenotype in Case 2. Abbreviations: ANA, antinuclear antibody; anti-dsDNA, anti-double-stranded DNA antibody; ANCA, antineutrophil cytoplasmic antibody; anti-GBM, anti-glomerular basement membrane antibody; ARB, angiotensin receptor blocker; C3, complement component 3; EM, electron microscopy; eGFR, estimated glomerular filtration rate; FGN, fibrillary glomerulonephritis; GBM, glomerular basement membrane; GN, glomerulonephritis; HBV/HCV, hepatitis B/C virus; IF, immunofluorescence; IFE, immunofixation electrophoresis; IFTA, interstitial fibrosis/tubular atrophy; PLA2R, phospholipase A2 receptor; RPGN, rapidly progressive glomerulonephritis; SPEP, serum protein electrophoresis; UTI, urinary tract infection.

Feature	Case 1	Case 2
Dominant phenotype	Acute severe kidney dysfunction/RPGN; hypertensive emergency-range blood pressure; edema; dialysis initiated	Chronic proteinuria; preserved eGFR; minimal urinary sediment
Clinical confounders	*Klebsiella pneumoniae* urinary tract infection; severe hypertension; mild bilateral hydronephrosis; recent amoxicillin/clavulanate exposure; acute tubulointerstitial injury; severe metabolic derangements	Positive anti-dsDNA/borderline ANA and thyroid autoimmunity; no acute infectious or obstructive trigger reported
Serology and immune workup	ANA speckled 2+; anti-dsDNA, ANCA, anti-GBM, anti-PLA2R, antiphospholipid antibodies, HIV, and hepatitis B negative; SPEP/IFE normal	Positive anti-dsDNA; borderline ANA; HBV/HCV, anti-PLA2R, SPEP/IFE, and serum free light chains negative or normal
Complement levels	Normal	Normal
Light microscopy	Crescentic immune-complex GN pattern; 11 cellular/fibrocellular crescents among 20 glomeruli; acute tubulointerstitial injury	Mesangial matrix expansion, mild mesangial hypercellularity, GBM thickening; tubulointerstitium unremarkable
Congo red staining	Negative	Negative
Immunofluorescence pattern	Immune-complex staining for IgG and C3 in mesangial and capillary wall distributions; C1q staining; both kappa and lambda light chain staining; polytypic pattern	Mesangial and capillary wall immune-complex staining with IgG and C3; C1q staining; both kappa and lambda light chain staining; polytypic pattern
IgG subclass staining	Not performed	Not performed
Electron microscopy availability and findings	Not performed because insufficient biopsy material remained for ultrastructural evaluation	Randomly arranged, nonbranching fibrillary deposits measuring approximately 13–19 nm.
DNAJB9 staining pattern	Positive	Positive
Chronicity/IFTA	Seven glomeruli showed ischemic changes or global sclerosis; tubular damage with dilatation and interstitial inflammation were present	Tubules, interstitium, and vessels were reported as unremarkable
Diagnostic interpretation	DNAJB9-positive crescentic immune-complex GN, interpreted as probable FGN because EM and IgG subclass staining were unavailable. Diagnostic certainty was lower than in Case 2. The dialysis-requiring outcome was substantially confounded by severe hypertension, infection, mild obstruction, and acute tubulointerstitial injury.	DNAJB9-positive FGN with chronic proteinuric phenotype and lupus-like serologic features. Classic active lupus nephritis was not supported by normal complement, inactive urinary sediment, absence of active proliferative lesions, EM-confirmed fibrils, and positive DNAJB9 staining
Proposed descriptive clinicopathologic interpretation	High inflammatory activity around DNAJB9/IgG/complement-containing deposits with crescent formation and rapid functional loss	Lower inflammatory activity or earlier/chronic deposit phenotype dominated by proteinuria and preserved filtration
Treatment	Pulse intravenous methylprednisolone 1 g daily for 3 days, followed by high-dose oral prednisone taper; three doses of intravenous cyclophosphamide, 900 mg per dose; maintenance hemodialysis	Supportive nephroprotective therapy with angiotensin receptor blockade and dapagliflozin; no immunosuppressive therapy
Follow-up and renal outcome	No renal recovery; remained dialysis-dependent on maintenance hemodialysis.	Proteinuria decreased to <1 g/day and kidney function remained preserved.

## Data Availability

The data supporting the findings of this study are not publicly available due to patient privacy and confidentiality considerations. De-identified data may be made available from the corresponding author upon reasonable request, subject to institutional and ethical requirements.

## References

[1] Rosenmann E., Eliakim M. (1977). Nephrotic syndrome associated with amyloid-like glomerular deposits. Nephron.

[2] Iskandar S.S., Falk R.J., Jennette J.C. (1992). Clinical and pathologic features of fibrillary glomerulonephritis. Kidney Int..

[3] Rosenstock J.L., Markowitz G.S., Valeri A.M., Sacchi G., Appel G.B., D’Agati V.D. (2003). Fibrillary and immunotactoid glomerulonephritis: Distinct entities with different clinical and pathologic features. Kidney Int..

[4] Nasr S.H., Valeri A.M., Cornell L.D., Fidler M.E., Sethi S., Leung N., Fervenza F.C. (2011). Fibrillary glomerulonephritis: A report of 66 cases from a single institution. Clin. J. Am. Soc. Nephrol..

[5] Sethi S., Theis J.D., Vrana J.A., Fervenza F.C., Sethi A., Qian Q., Quint P., Leung N., Dogan A., Nasr S.H. (2013). Laser microdissection and proteomic analysis of amyloidosis, cryoglobulinemic GN, fibrillary GN, and immunotactoid glomerulopathy. Clin. J. Am. Soc. Nephrol..

[6] Dasari S., Alexander M.P., Vrana J.A., Theis J.D., Mills J.R., Negron V., Sethi S., Dispenzieri A., Highsmith W.E., Nasr S.H. (2018). DNAJ heat shock protein family B member 9 is a novel biomarker for fibrillary GN. J. Am. Soc. Nephrol..

[7] Andeen N.K., Yang H.Y., Dai D.F., MacCoss M.J., Smith K.D. (2018). DNAJ homolog subfamily B member 9 is a putative autoantigen in fibrillary GN. J. Am. Soc. Nephrol..

[8] Nasr S.H., Vrana J.A., Dasari S., Bridoux F., Fidler M.E., Kaaki S., Quellard N., Rinsant A., Goujon J.M., Sethi S. (2018). DNAJB9 is a specific immunohistochemical marker for fibrillary glomerulonephritis. Kidney Int. Rep..

[9] Kudose S., Canetta P., Andeen N.K., Stokes M.B., Batal I., Markowitz G.S., D’Agati V.D., Santoriello D. (2021). Diagnostic approach to glomerulonephritis with fibrillar IgG deposits and light chain restriction. Kidney Int. Rep..

[10] Andeen N.K., Kung V.L., Robertson J., Gurley S.B., Avasare R.S., Sitaraman S. (2022). Fibrillary glomerulonephritis, DNAJB9, and the unfolded protein response. Glomerular Dis..

[11] Klomjit N., Alexander M.P., Zand L. (2020). Fibrillary glomerulonephritis and DNAJ homolog subfamily B member 9 (DNAJB9). Kidney360.

[12] Andeen N.K., Troxell M.L., Riazy M., Avasare R.S., Lapasia J., Jefferson J.A., Akilesh S., Najafian B., Nicosia R.F., Alpers C.E. (2019). Fibrillary glomerulonephritis: Clinicopathologic features and atypical cases from a multi-institutional cohort. Clin. J. Am. Soc. Nephrol..

[13] Said S.M., Rocha A.B., Royal V., Valeri A.M., Larsen C.P., Theis J.D., Vrana J.A., McPhail E.D., Bandi L., Safabakhsh S. (2021). Immunoglobulin-negative DNAJB9-associated fibrillary glomerulonephritis: A report of 9 cases. Am. J. Kidney Dis..

[14] Said S.M., Leung N., Alexander M.P., Cornell L.D., Fidler M.E., Grande J.P., Herrera L.H., Sethi S., Zhang P., Nasr S.H. (2020). DNAJB9-positive monotypic fibrillary glomerulonephritis is not associated with monoclonal gammopathy in the vast majority of patients. Kidney Int..

[15] Avasare R.S., Robinson B.A., Nelson J., Woltjer R., Krajbich V., Nguyen V., Garcia D., Setthavongsack N., Kizzar C., Raess P.W. (2020). DNAJB9 is not transcriptionally upregulated in the glomerulus in fibrillary glomerulonephritis. Kidney Int. Rep..

[16] Nasr S.H., Dasari S., Lieske J.C., Benson L.M., Vanderboom P.M., Holtz-Heppelmann C.J., Giesen C.D., Snyder M.R., Erickson S.B., Fervenza F.C. (2019). Serum levels of DNAJB9 are elevated in fibrillary glomerulonephritis patients. Kidney Int..

[17] Javaugue V., Said S.M., Karras A., Bu L., Bridoux F., François A., Goujon J.-M., Fayad R., Ross D., Sastry A. (2023). Prognostic value of diffuse crescentic lesions in fibrillary glomerulonephritis. Am. J. Kidney Dis..

[18] Patrick J., Charles-Rudwick M., Quinn C., Paterson A., Soe L.T., Varma R., Swift O., Glancey G., Galahitiyawa C., Wilcocks L. (2025). Fibrillary glomerulonephritis: An observational study of clinical-pathological features and outcomes in patients from a multi-institutional cohort. Clin. Kidney J..

[19] Attieh R.M., Yang Y., Rosenstock J.L. (2024). Updates on the diagnosis and management of fibrillary glomerulonephritis. Adv. Kidney Dis. Health.

[20] Hogan J., Restivo M., Canetta P.A., Herlitz L.C., Radhakrishnan J., Appel G.B., Bomback A.S. (2014). Rituximab treatment for fibrillary glomerulonephritis. Nephrol. Dial. Transplant..

[21] Erickson S.B., Zand L., Nasr S.H., Alexander M.P., Leung N., Drosou M.E., Fervenza F.C. (2021). Treatment of fibrillary glomerulonephritis with rituximab: A 12-month pilot study. Nephrol. Dial. Transplant..

